# Outcomes of Superior Capsular Reconstruction Using the Long Head of the Biceps Tendon in Large to Massive Rotator Cuff Tears: A Meta-Analysis and Systematic Review

**DOI:** 10.3390/jcm13041052

**Published:** 2024-02-12

**Authors:** Kyun-Ho Shin, Il-Tae Jang, Seung-Beom Han

**Affiliations:** 1 Department of Orthopedic Surgery, Yeson Hospital, Bucheon-si 14555, Republic of Korea; 2 Nanoori Medical Research Institute, Seoul 06048, Republic of Korea; nanoori_research@naver.com; 3 Department of Orthopedic Surgery, Anam Hospital, Korea University College of Medicine, Seoul 02841, Republic of Korea; oshan@korea.ac.kr

**Keywords:** shoulder joint, arthroscopy, rotator cuff, rotator cuff injuries, biceps brachii, biceps tendon, superior capsular reconstruction

## Abstract

(1) Background: Addressing large to massive rotator cuff tears (LMRCTs) poses complex challenges. This systematic review investigated outcomes of superior capsular reconstruction (SCR) with the long head of the biceps tendon (LHBT) compared to conventional rotator cuff repair (RCR) for LMRCTs. (2) Methods: A systematic search across the MEDLINE/PubMed, EMBASE, Cochrane Library, and Scopus databases until 1 October 2023 identified studies that directly compared SCR with LHBT with conventional RCR in patients with LMRCTs and included a minimum of a 12-month follow-up period. The assessed outcome measures encompassed retear rates, functional outcomes, range of motion (ROM), and acromiohumeral interval (AHI). Risk of bias assessment was conducted via the Robins-I tool. (3) Results: In six studies with 456 cases (210 SCR using LHBT and 246 using RCR), SCR with LHBT significantly reduced retear rates (OR = 0.21; 95% CI, 0.12–0.36; *p* < 0.01; I^2^ = 0%). Furthermore, SCR with LHBT showed significant improvement in range of forward flexion (SMD 0.32, 95% CI: 0.09–0.55, *p* < 0.01, I^2^ = 39%) and AHI (SMD 0.61, 95% CI: 0.31–0.92, *p* < 0.01, I^2^ = 0%) postoperatively. (4) Conclusion: SCR with LHBT is a safe and effective treatment for LMRCTs, reducing retear rates, maintaining greater postoperative AHI, and improving ROM compared to conventional RCR. Additional high-quality interventional studies are needed to confirm these results.

## 1. Introduction

Rotator cuff tears significantly affect individuals’ quality of life, often leading to substantial functional limitations [1,2]. Managing large to massive rotator cuff tears (LMRCTs) poses complex challenges. While conservative treatments offer temporary relief, they often result in persistent pain and rotator cuff arthropathy. Surgical repair for LMRCTs is complicated by issues such as tendon retraction, fatty infiltration, and atrophy, all hindering the natural healing process [3,4,5,6]. It is crucial to note that surgically repaired LMRCTs can fail structurally in as many as 94% of cases [3,4,5,6,7,8].

Various surgical techniques have been proposed for LMRCT management, including medialized repair, partial repair, patch augmentation, tendon transfer, reverse total shoulder arthroplasty, subacromial resurfacing, bursal acromial reconstruction, and subacromial balloon spacer placement [9,10,11]. Among these, superior capsular reconstruction (SCR), introduced by Mihata et al., has demonstrated promising outcomes by preventing static superior migration of the humeral head [12,13]. Recently, the long head of the biceps tendon (LHBT) has emerged as an alternative to standard SCR grafts. SCR using LHBT has been described in various ways, such as biceps rerouting, biceps transposition, superior capsular augmentation, and anterior cable reconstruction [14,15,16,17,18,19]. This method involves preserving the LHBT’s natural attachment on the glenoid side and moving the proximal part backward, making it biomechanically comparable to SCR using a tensor fascia lata (TFL) autograft [10,20]. Notably, this technique not only offers a local autologous source of collagen graft but also enables entirely arthroscopic procedures, minimizing donor site morbidity [14,15,16,17,18,19].

Despite optimistic reports on the effectiveness of SCR using LHBT for LMRCTs, previous systematic reviews were limited by the inclusion of case series studies without meta-analyses [14,21,22,23]. Additionally, while SCR using LHBT offers several advantages, it is important to acknowledge potential drawbacks, including a longer operation time. Importantly, several recently published high-quality comparative studies have provided valuable insights into the effects of SCR using LHBT in LMRCTs. These additional studies contribute to a deeper understanding of the impact of SCR using LHBT in LMRCTs [17,18,19,24,25]. This meta-analysis aims to comprehensively review, summarize, and analyze existing clinical data related to SCR using LHBT for LMRCTs. We hypothesize that SCR using LHBT will lead to significantly improved outcomes, including reduced retear rates and enhanced functional results compared to conventional rotator cuff repair (RCR) for LMRCTs. A meticulous evaluation of this research will empower clinicians, enabling them to make well-informed decisions regarding the optimal surgical technique for treating LMRCTs.

## 2. Materials and Methods

This study adhered to the guidelines outlined in the Preferred Reporting Items for Systematic Reviews and Meta-Analyses (PRISMA) to ensure accuracy and transparency in reporting [26]. This study is registered with the Research Registry, and the unique identifying number is reviewregistry1766. The tasks, including study screening, selection, quality assessment, data extraction, and result pooling, were independently conducted by two authors. Discrepancies were resolved through consultation with a third independent author to ensure consistency and accuracy. 

### 2.1. Search Strategy

A comprehensive search was conducted on 1 October 2023 across prominent databases, including MEDLINE/PubMed, Cochrane Library, Embase, and Scopus. The search terms, combining keywords, medical subject heading (MeSH) terms, and their variations within the [Title/Abstract] field, included “rotator cuff”, “supraspinatus”, “infraspinatus”, “subscapularis”, “teres minor”, “rotator cuff” [MeSH term], “rotator cuff injuries” [MeSH term], and “rotator cuff arthropathy” [MeSH term], along with “long head of biceps”, “biceps long head”, or “long head of the biceps tendon”. Our search was linguistically restricted to studies conducted in English. The impact of the language restriction on bias was minimized, given the consensus in the literature that restriction to English-language studies in systematic reviews does not significantly affect overall information [27]. Additionally, the reference lists of selected articles were scrutinized to identify potentially overlooked relevant studies.

### 2.2. Inclusion and Exclusion Criteria

Inclusion criteria:

1. Participants: The study included patients who underwent primary arthroscopic surgery for LMRCTs.
2. Interventions: Patients in the SCR-BT group received arthroscopic SCR using LHBT for LMRCTs (SCR group).
3. Comparisons: The control group (RCR group) underwent arthroscopic conventional RCR for LMRCTs.
4. Primary outcomes: The study assessed various outcomes, including rotator cuff retear rates (evaluated through magnetic resonance imaging or sonography), functional outcomes measured by the Constant score, American Shoulder and Elbow Surgeons (ASES) score, University of California at Los Angeles (UCLA) score, and Simple Shoulder Test (SST) score.
5. Secondary outcomes: Secondary outcomes included the visual analog scale (VAS), postoperative range of motion (ROM), and strength, and radiological outcomes included acromiohumeral interval (AHI).
6. Follow-up: The included studies required a minimum clinical follow-up of 12 months.

Exclusion criteria:

Studies that did not meet the inclusion criteria outlined above were excluded. Non-comparative studies, case reports, and studies lacking relevant outcome measures were also excluded. Studies that did not directly compare SCR using LHBT with conventional RCR for LMRCTs were excluded from the analysis.

### 2.3. Study Screening, Data Collection and Quality Assessment

During the first stage of screening, duplicated publications were removed, and two independent authors screened all the titles and abstracts. The full texts of the articles were reviewed in the second stage of the screening process to select articles according to the inclusion and exclusion criteria. Data extraction was performed by two independent authors based on the descriptive information obtained from the selected studies. The extracted data encompassed the following aspects: (1) study characteristics, such as the name of the first author, publication year, and country; (2) patient demographics, including the number of patients, sex, and age; (3) details regarding the size, characteristics, and reparability of rotator cuff tendon tears; (4) surgical procedures, including the specific repair method employed and any concomitant procedures performed; (5) information regarding implemented rehabilitation programs; (6) duration of the follow-up period; and (7) primary and secondary outcomes. The Robins-I tool was used to assess risk of bias in non-randomized studies of interventions [28]. Visual plots representing the risk of bias were generated using *robvis* [29].

### 2.4. Statistical Analysis

Inter-reviewer reliability was assessed using the kappa statistic (κ) for study screening and selection, quality evaluation, data extraction, and result pooling. For continuous outcomes, the intergroup difference in mean outcomes divided by the standard deviation of the difference was calculated as the standardized mean difference (SMD) and presented along with a 95% confidence interval (CI). Dichotomous outcomes were analyzed using the odds ratio (OR) with a 95% CI. Meta-analyses were conducted to combine the effects and calculate corresponding 95% CIs. The presence of heterogeneity was assessed through a test of homogeneity based on the χ^2^ test, I^2^ statistics, and Q statistics. If I^2^ statistics were less than 50%, indicating low heterogeneity, the fixed-effect model (Mantel–Haenszel method) was applied. In cases where I^2^ was 50% or higher, indicating significant heterogeneity, a “leave-one-out” sensitivity analysis was conducted to identify the potential source of heterogeneity. If heterogeneity persisted even after excluding each study, the random-effects model (DerSimonian–Laird method) was utilized [30]. Subgroup analyses were performed for available outcomes based on SCR techniques including dynamic SCR using LHBT without distal tenotomy or static SCR using distally tenotomized LHBT. Publication bias was assessed using Egger’s regression symmetry test [31]. All statistical analyses were conducted using Rstudio v.1.0.143 (RStudio Inc., Boston, MA, USA), with a significance level set at *p* < 0.05.

## 3. Results

### 3.1. Search Results

**Figure 1:**
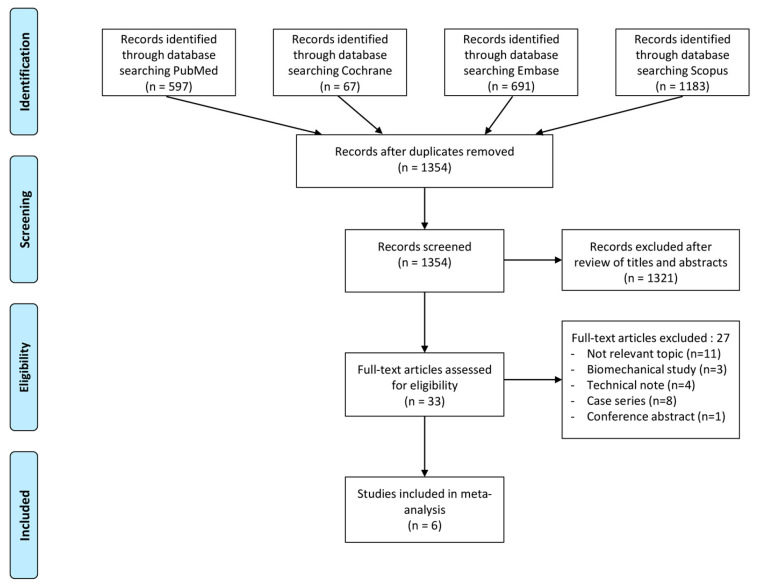
PRISMA flow diagram.

Figure 1 provides an overview of the process used to identify and select studies for inclusion. Initially, a total of 2538 articles were identified through the literature search. After removing 1184 duplicates, the remaining 1354 articles underwent screening based on their titles and abstracts. Subsequently, 33 full-text articles were assessed for eligibility. Out of these, 27 articles were excluded, as they did not meet the predetermined inclusion criteria. Ultimately, six articles were included in the meta-analysis [7,17,18,19,24,25]. The reliability values for tasks such as study screening, selection, quality evaluation, data extraction, and result pooling ranged from 0.95 to 1.00, indicating an excellent level of agreement.

### 3.2. Study Characteristics 

Table 1 presents the baseline characteristics of the studies included in this meta-analysis. A total of 456 patients, comprising 210 patients who underwent arthroscopic SCR using LHBT and 246 patients who underwent arthroscopic conventional RCR for LMRCTs, were included. The mean age of the patients ranged between 60.0 and 71.9 years, and the mean follow-up period ranged from 15.1 to 37.2 months. Four studies [17,18,19,24] enrolled patients with large to massive, full-thickness rotator cuff tears, while two studies [7,25] included patients with massive rotator cuff tears involving ≥2 tendons. The repair techniques and postoperative rehabilitation programs are summarized in Table 2.

### 3.3. Quality Assessment

**Figure 2:**
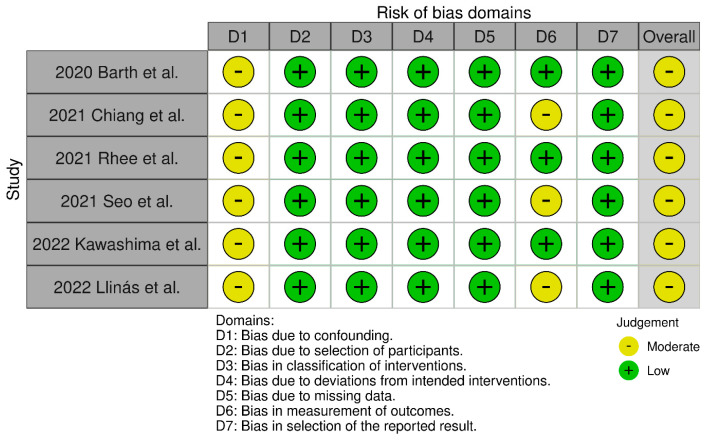
Risk of bias per domain and overall within the included studies.

**Figure 3:**
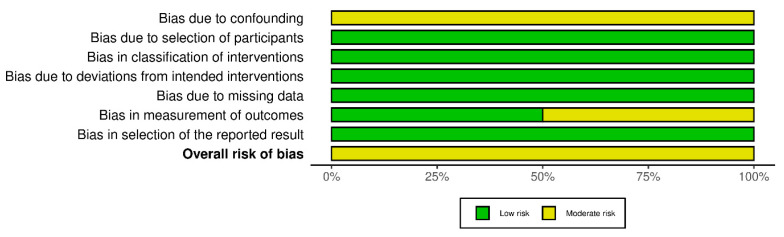
Summary of the risk of bias per domain and overall.

The evaluation of risk of bias in the included studies is depicted in Figure 2 and Figure 3. All studies were identified to carry a risk of time-varying confounding. Additionally, three studies had an unclear risk of bias in outcome measurement [19,24,25]. Overall, all studies were categorized as having a moderate risk of bias.

### 3.4. Meta-Analysis Results

#### Retear

**Figure 4:**
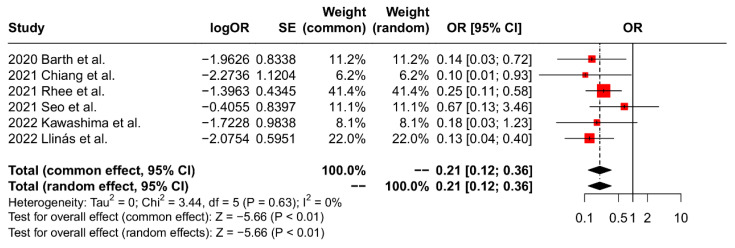
Forest plot of postoperative retear rates.

The findings of the meta-analyses regarding retear rates are summarized in Table 3. All studies included in the analysis reported the retear rate, which was evaluated using either postoperative MRI or sonography. The results of the pooled analysis demonstrated a significant reduction in the incidence of postoperative retears in the SCR group when compared to the RCR group (OR 0.21, 95% CI: 0.12 to 0.36, *p* < 0.01, I^2^ = 0%; Figure 4).

### 3.5. Clinical and Radiological Outcomes

**Figure 5:**
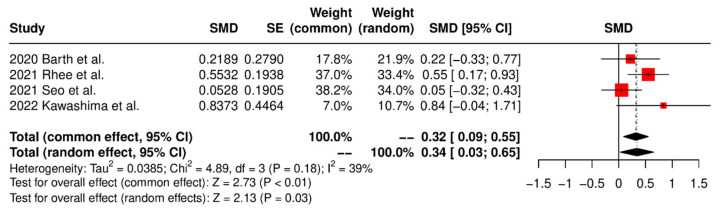
Forest plot of range of forward flexion.

**Figure 6:**
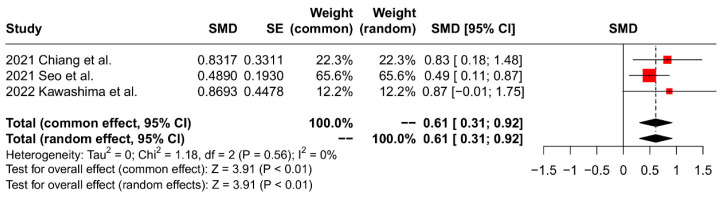
Forest plot of AHI.

The clinical outcomes are summarized in Table 3. Two studies [7,17] assessed the total Constant score, while two studies [17,18] evaluated the UCLA score postoperatively. The meta-analysis revealed no significant intergroup difference for the total Constant score (SMD 0.02, 95% CI: −0.29 to 0.32, *p* = 0.92, I^2^ = 0%) and UCLA score (SMD 0.23, 95% CI: −0.52 to 0.99, *p* = 0.55, I^2^ = 64%). Five studies examined the ASES score postoperatively [7,17,18,19,25]. The pooled results showed no significant intergroup difference for the postoperative ASES score (SMD 0.05, 95% CI: −0.28 to 0.18, *p* = 0.65, I^2^ = 0%). Four studies examined postoperative VAS, and the meta-analysis results indicated no significant intergroup difference (SMD 0.10, 95% CI: −0.30 to 0.11, *p* = 0.35, I^2^ = 38%) [7,17,19,25]. Four studies [7,17,18,19] reported range of forward flexion, while five studies [7,17,18,19,25] reported range of external rotation postoperatively. The pooled results showed a significant intergroup difference in favor of the SCR group for the range of forward flexion (SMD 0.32, 95% CI: 0.09 to 0.55, *p* < 0.01, I^2^ = 39%; Figure 5). However, there was no significant intergroup difference in the range of external rotation (SMD −0.10, 95% CI: −0.29 to 0.10, *p* = 0.34, I^2^ = 28%). Furthermore, four studies evaluated the postoperative AHI [17,18,19,24]. The pooled results showed a significant intergroup difference in favor of the SCR group for AHI (SMD 0.61, 95% CI: 0.31 to 0.92, *p* < 0.01, I^2^ = 0%; Figure 6).

### 3.6. Subgroup Analyses

The details of the sensitivity and subgroup analyses are summarized in Supplementary Table S1. The statistical results were stable and supported our conclusion favorably. Subgroup analyses found no significant statistical differences according to SCR technique in terms of retear risk, VAS, ASES score, ROM, and AHI postoperatively.

### 3.7. Sensitivity Analyses

Significant heterogeneity was observed in the pooled results of postoperative ASES score. Sensitivity analyses highlighted the study conducted by Lilnas et al., as a potential source of this heterogeneity [25]. As a result, this study was excluded from the meta-analysis, as shown in Supplementary Figures S1 and S2. Furthermore, significant heterogeneity was observed in the pooled results of postoperative AHI. Sensitivity analyses highlighted the study conducted by Rhee et al., as a potential source of this heterogeneity [17]. As a result, this study was excluded from the meta-analysis, as shown in Supplementary Figures S3 and S4.

## 4. Discussion

Our study highlights the efficacy of SCR with LHBT in reducing retear rates during rotator cuff repair, outperforming conventional RCR. Additionally, SCR with LHBT not only significantly decreased humeral head superior translation but also showcased improved postoperative ROM compared to conventional RCR.

The challenging landscape of managing LMRCTs is evident, with reported retear rates soaring up to 94% [3,4,5,32]. As the success of RCR is intricately linked to the structural integrity of the repaired tendon, LMRCTs often result in less favorable prognoses. Complications such as joint stiffness or cuff tear arthropathy development over time, often accompanied by humeral head upmigration, further complicate matters [32,33]. Within the spectrum of LMRCT treatment options, SCR has emerged as a viable choice [12,13]. Recent biomechanical studies utilizing cadaver models have confirmed that SCR with LHBT graft provides sufficient failure strength compared to SCR using a tensor fascia lata autograft [10,20,34,35]. Consequently, various surgical techniques using LHBT grafts for SCR have been introduced and are gaining considerable attention [14,15,16,17].

Therefore, this review aims to offer a comprehensive summary and synthesis of the current evidence on the surgical outcomes of SCR using autogenous LHBT compared to arthroscopic conventional RCR. Specifically, this review addresses three key questions: (1) “Does it improve retear rates or tendon integrity?”; (2) “Does it prevent upmigration of the humeral head?”; and (3) “Does it enhance functional outcomes?”.

Our study findings strongly support the initial hypothesis that SCR using LHBT for LMRCTs is linked to reduced retear rates and the prevention of humeral head upmigration compared to arthroscopic conventional RCR. The lower retear rates and increased AHI observed in the SCR using LHBT group may be attributed to the action of autogenous tenocytes in the LHBT and the augmented biceps tendon, potentially providing an additional blood supply that promotes tendon-to-bone healing in the rotator cuff tendon [36,37,38]. Furthermore, in their report, Mihata et al., emphasized that the success of SCR, regardless of the graft type used, primarily depends on the final graft and cuff tendon healing [39,40]. Their findings suggest that SCR contributes to the preservation of the force couple of the shoulder joint in both the coronal and horizontal planes, ultimately preventing possible proximal migration of the humeral head. This preservation results in an increased AHI and reduced retear rates.

The survival of the SCR graft is a critical determinant of clinical outcomes [12,13,40]. When utilizing grafts such as dermal allograft or fascia lata autograft, reported graft failure rates vary widely, ranging from 3.4% to 90.0% [12,40,41,42,43,44]. Recent reports indicate that the use of fascia lata autograft in patients with pseudoparalysis resulted in a high graft healing rate of 95% [40]. Although all patients experienced significant increases in active elevation and external rotation, two patients with residual pseudoparalysis postoperatively encountered graft tears [40]. However, there is still limited reporting on the graft survival of SCR using LHBT. Chiang et al., reported a 1-year follow-up graft failure rate of 5.6% for SCR using LHBT, and even in the cases of failure, satisfactory clinical outcomes were achieved without critical complaints [24]. Rhee et al., highlighted the impact of the quality of the biceps tendon and the extent of the tear on the retear of the rotator cuff and graft survival [17]. In SCR using LHBT with partial tears involving more than 50% of the tendon, the postoperative retear rate of the rotator cuff tendon was as high as 35.7% [17]. Further research is warranted to understand under what conditions the biceps tendon is suitable for use as an SCR graft and how the fixation method influences graft survival.

Despite the reduction in humeral head upmigration and the retear rate of the rotator cuff tendon, SCR using LHBT resulted in only a slight improvement in the postoperative range of forward flexion. However, there was no statistically significant difference in functional outcome measures. The assumption that partial repair of a massive rotator cuff tear might improve the biomechanics of the shoulder while re-establishing essential force couples, thereby converting a dysfunctional symptomatic rotator cuff tear into a functional tear, guided our approach. Moreover, complete closure of the defect was not considered essential for restoring normal rotator cuff biomechanics. In the present study, partial tendon repair for anterior L-shaped rotator cuff tears was attempted to reduce the exposed footprint area after repair and restore essential force couples by advancing posteromedial supraspinatus and infraspinatus tendons [3,45,46,47,48]. Despite efforts, the limited sample size and short-term follow-up may affect the demonstration of statistical significance in functional outcomes related to retear rates. Barth et al., reported an increase in postoperative strength, with the healed tendon group outperforming the structural failure group in Constant, ASES, SST, SSV, VAS, and strength measures [7]. In a study with a relatively longer follow-up, Llinás et al., observed improved functional outcomes, reporting superior ROM, ASES, and VAS in the SCR group [25]. Additionally, Kawashima et al., demonstrated the superiority of the SCR group, with a higher number of patients meeting the threshold for clinically meaningful outcomes, including minimal clinically important difference, substantial clinical benefit, and patient-acceptable symptomatic state [18]. The primary objective of SCR is not only to enhance functional outcomes but also to prevent long-term cuff tendon retears and cuff tear arthropathy progression. This study, by demonstrating the prevention of humeral head upmigration and reduced rotator cuff retear rates compared to conventional RCR, affirms the utility of SCR using LHBT in large to massive rotator cuff tears.

This review exhibits both strengths and limitations. This review stands as a pioneering comprehensive meta-analysis, focusing on SCR using LHBT for the treatment of LMRCTs. It meticulously synthesizes available evidence, offering invaluable insights into the effectiveness of SCR with LHBT. Rigorous subgroup analyses categorized by SCR technique add robustness to the results. These strengths deepen our understanding of the optimal surgical approach for LMRCTs, providing evidence-based guidance for clinical decisions.

Nonetheless, this review is not without limitations. First, the analysis was confined to a limited number of studies characterized by short-term follow-up periods and low levels of evidence. Secondly, substantial heterogeneity existed among the studies in terms of tear size, repair techniques, rehabilitation protocols, and outcome assessment methods, introducing a level of variability that necessitates caution in interpretation. Although subgroup analyses based on SCR techniques, encompassing SCR with LHBT with or without distal tenotomy, have demonstrated robust results, the findings should be interpreted with caution. Thirdly, the study faced limitations such as the inability to compare outcomes with those of other procedures or grafts, underscoring the need for future studies comparing SCR groups with different grafts for a more comprehensive understanding. Kim et al., reported comparable outcomes between SCR using BT autograft and SCR using HD allograft tissue, with no significant difference noted [49]. Similarly, a previous study comparing TFL and LHBT grafts in SCR found comparable results [23]. Future studies comparing SCR groups with different grafts are needed for a more comprehensive understanding. Fourthly, inconsistent documentation and analysis of potential risk factors and confounding variables, such as smoking, body mass index, diabetes, and augmentation techniques, limits definitive conclusions about their impact on outcomes [50,51,52,53,54,55]. Previous studies comparing patch augmentation with the SCR group yielded conflicting results [7,15]. Comparative studies considering multiple variables are needed for a comprehensive understanding. Fifthly, the focus on short-term outcomes due to limited long-term data underscores the need for well-designed randomized, controlled trials with larger sample sizes, longer-term follow-up, and better control of confounding factors to establish conclusive evidence.

## 5. Conclusions

SCR with LHBT is a safe and effective treatment for LMRCTs, reducing retear rates, maintaining higher postoperative AHI, and improving ROM compared to conventional RCR. The technique is advantageous for its safety, ease of use, time-saving nature, and cost-effectiveness, with the primary precondition being a relatively intact LHBT. Additional high-quality interventional studies are needed to confirm these results.

## Figures and Tables

**Table 1:** Characteristics of included studies.

First Author (Year)	Study Design	Country	Level of Evidence	Sample Size (n)		Mean Age (Years)		Male n (%)		Follow Up		Type of Injury	Outcome Measurement
				SCR with LHBT	Conventional RCR	SCR with LHBT	Conventional RCR	SCR with LHBT	Conventional RCR	SCR with LHBT	Conventional RCR		
Barth et al., (2020) [7]	Retrospective case–control study	France	III	24	28	60	63	16 (66.7)	15 (53.6)	Mean 25 (SD 2) months (minimum 24 months)	Mean 25 (SD 2) months (minimum 24 months)	Symptomatic massive posterosuperior RCTs involving ≥2 tendons; retracted tendon and/or poor tissue quality involving >50% of the supraspinatus and infraspinatus footprint	Retear rate at 12 months, VAS, ASES, SST, Constant, and ROM
Chiang et al., (2021) [24]	Retrospective case–control study	Taiwan	III	18	22	62.3	62.2	7 (38.9)	6 (27.3)	Mean 26.6 (SD 3.9) months (minimum 24 months)	Mean 31.9 (SD 6.4) months (minimum 24 months)	Large to massive reparable RCTs (≥3 cm)	Retear rate at 12 months, VAS, ASES, UCLA, ROM, and AHI
Rhee et al., (2021) [17]	Retrospective case–control study	Republic of Korea	III	59	52	63.7	62.8	32 (54.2)	29 (55.8)	Mean 15.1 (SD 3.4) months (minimum 12 months)	Mean 25.1 (SD 8.7) months (minimum 12 months)	Large (3–5 cm) to massive (>5 cm) RCTs, as measured intraoperatively	Retear rate at 12 months, Constant, ASES, UCLA, VAS, ROM, and AHI
Seo et al., (2021) [19]	Retrospective case–control study	Republic of Korea	III	41	84	61.9	62.6	18 (31.7)	36 (42.9)	Mean 18.7 (SD 4.2) months (minimum 12 months)	Mean 15.6 (SD 2.1) months (minimum 12 months)	Large to massive RCTs confirmed on preoperative MRI as more than a 2 cm tear size and anterior cable disruption (it was determined that a disruption of the anterior rotator cable was present if the supraspinatus tendon was completely detached from the anterior attachment site and the LHBT along the bicipital groove was exposed in the subacromial space during arthroscopy)	Retear rate at 12 months, ASES, VAS, ROM, and AHI
Kawashima et al., (2022) [18]	Retrospective case–control study	Japan	III	12	10	67.8	71.9	7 (58.3)	6 (60.0)	Mean 24.8 (Range 24–30) months (minimum 24 months)	Mean 37.2 (Range 24–72) months (minimum 24 months)	Irreparable large to massive RCTs with inability of the tendon to reach the original footprint after significant releases of the capsule and subdeltoid synovial tissue	Retear rate at 24 months, ASES, UCLA, ROM, and AHI
Llinás et al., (2022) [25]	Retrospective case–control study	Colombia	III	56	50	62.2	64.2	35 (62.5)	36 (72)	Mean 26 (SD 1.55) months (minimum 24 months)	Mean 27.47 (SD 2.6) months (minimum 24 months)	>2.5 cm exposure of the GT footprint in the anteroposterior plane seen during arthroscopy Retraction of the supraspinatus tendon to at least the humeral head (Patte classification grades 2 and 3) and fatty infiltration of the supraspinatus muscle belly (Goutallier classification grades 3 and 4)	Retear rate at 24 months, ASES, VAS, and ROM

SCR, superior capsular reconstruction; LHBT, long head of the biceps tendon; RCR, rotator cuff repair; SD, standard deviation; RCT, rotator cuff tear; VAS, visual analog scale; ASES, the American Shoulder and Elbow Surgeons score; SST, simple shoulder test; ROM, range of motion; UCLA, the University of California at Los Angeles score; AHI, acromiohumeral interval.

**Table 2:** Techniques of SCR with LHBT and conventional RCR and postoperative rehabilitation.

First Author (Year)	Techniques of SCR with LHBT	Conventional RCR Technique	Concomitant Procedures	Rehabilitation			
				Immobilization	Passive Motion Exercises	Active Motion Exercises	Strengthening Exercises and Sports Activities
Barth et al., (2020) [7]	The LHBT autograft was tenotomized and fixed onto the greater tuberosity using a 5.5 mm triple-loaded suture anchor, which was inserted at the midpoint of the humeral greater tuberosity; Side-to-side suture (yellow arrows) of the LHBT with the infraspinatus; The distal part of the LHBT was left free without additional tenodesis.	RCR using DR repair technique	Biceps tenodesis high in the bicipital groove in the RCR group, as well as bursectomy and acromioplasty	Immobilization using a sling for 6 weeks postoperatively	Range-of-motion exercise program from 4 weeks postoperatively (passive, active assisted, and active motion)		From 6 months postoperatively
Chiang et al., (2021) [24]	The LHBT autograft was transposed and fixed onto the greater tuberosity using a double-loaded suture anchor, which is placed into the middle (anteroposterior aspect) and medial (mediolateral aspect) of the prepared footprint in the shoulder placed at a 30° abduction and 10° forward flexion, with the elbow placed in a 90° flexion position; The tenotomy of the LHBT was performed about 1 to 1.5 cm distal to the fixation; RCR was performed superiorly to SCR using either SR, DR, or SB techniques.	RCR using either SR, DR, or SB repair technique	Biceps tenotomy in the RCR group, as well as bursectomy and acromioplasty	Immobilization using a sling for 4 weeks postoperatively	From 4 week postoperatively	From 12 weeks postoperatively after immobilization period	Sports activities requiring overhead motion from 6 months postoperatively
Rhee et al., (2021) [17]	The transverse humeral ligament and the biceps pulley were incised around the bicipital groove to mobilize the LHBT Using a 4 mm spherical burr, a 3 mm deep “neogroove” was created for the tendon at a new location 1 to 1.5 cm posterior to the lateral edge of the bicipital groove; Onlay biceps tenodesis was performed using a double-loaded suture anchor, which was fixed to the lateral humeral cortex 1 cm inferior to the neo-groove; Suture anchors for RCR were placed into the footprint immediately anterior and posterior to the newly positioned LHBT in its neogroove, and RCR was performed using the SR technique.	RCR using SR repair technique	Biceps tenotomy or tenodesis if the LHBT findings correlated with the clinical symptoms and signs in the RCR group, as well as bursectomy and acromioplasty	Immobilization using a sling for 4 weeks postoperatively	Passive range-of-motion exercises were allowed immediately.	From 8 weeks postoperatively after immobilization period	Progressive strengthening exercises from 8 weeks postoperatively and sports activities allowed from 6 months postoperatively
Seo et al., (2021) [19]	Biceps tenodesis was performed on the anterior greater tuberosity at the 1 cm superior aspect of the bicipital groove using an all-suture anchor; Owing to shifting and fixing of the LHBT at the anchor site, the LHBT from the anchor to the glenoid attachment site served as an ACR; RCR was performed using a standard SB technique with medial-row knot tying.	RCR using SB repair technique with medial-row knot tying	Subacromial decompression including acromioplasty	Immobilization using a sling for 4 weeks postoperatively	Passive range-of-motion exercises were allowed immediately.	From 4 to 8 weeks postoperatively after immobilization period	Progressive resistive exercises from 8 weeks postoperatively and sports activities allowed from 6 months postoperatively
Kawashima et al., (2022) [18]	The transverse humeral ligament was released, and the LHBT was transposed from the bicipital groove to the superior facet of the greater tuberosity; Two anchors with triple-loaded sutures were used for dual-row fixation. The medial anchor was placed at the edge of the articular cartilage medial to the highest point. The lateral anchor was placed 5 to 10 mm posterior to the bicipital groove at the superior facet; The LHBT was fixed onto the greater tuberosity in the shoulder placed at a 30° abduction and 15° external rotation; After the proximal part of the LHBT was secured in a new location, the distal part of the tenotomized LHBT was fixed to the proximal humerus for suprapectoral biceps tenodesis; Partial repair of superior rotator cuff tears was performed using the SR repair technique.	RCR using SR repair technique	Subacromial decompression and microfracture on the footprint immediately lateral to the fixation points with suture anchors	Immobilization using a sling for 5 weeks postoperatively	From 6 weeks postoperatively	From 6 weeks postoperatively	From 12 weeks postoperatively
Llinás et al., (2022) [25]	The transverse humeral ligament was released, and a double- or triple-loaded all-suture anchor was placed at the center of the rotator cuff footprint; Biceps tenotomy was performed distal to the tuberosity fixation; A side-to-side suture repair of the rotator cuff tear, incorporating the LHBT into the tear, was performed using one or two additional anchors with an SR or DR repair technique.	RCR using either SR or DR repair technique	Biceps tenotomy in the RCR group, as well as bursectomy, acromioplasty, and distal clavicle excision if needed	Immobilization using a sling for 4 weeks postoperatively	From 4 weeks postoperatively	From 4 weeks postoperatively	Sports activities were allowed from 6 months postoperatively

SCR, superior capsular reconstruction; LHBT, long head of the biceps; RCR, rotator cuff repair; DR, double row; SR, single row; SB, suture bridge.

**Table 3:** Summary of postoperative retear rates and functional outcomes between the SCR and RCR groups.

	OR or SMD	LL 95% CI	UL 95% CI	*p* Value	Number of Studies	Heterogeneity (%)	Analysis Model	Egger’s Test (*p* Value)
Retear rate	0.21	0.12	0.36	<0.01	6	0	Fixed	0.77
VAS	0.10	−0.30	0.11	0.35	4	38	Fixed	0.93
Constant score	0.02	−0.29	0.32	0.92	2	0	Fixed	NA
ASES score	0.05	−0.28	0.18	0.65	4	0	Fixed	0.50
UCLA score	0.23	−0.52	0.99	0.55	2	64	Random	NA
ROM (forward flexion)	0.32	0.09	0.55	<0.01	4	39	Fixed	0.56
ROM (external rotation)	−0.10	−0.29	0.10	0.34	5	28	Fixed	0.76
Acromiohumeral interval	0.61	0.31	0.92	<0.01	3	0	Fixed	0.19

SCR, superior capsular reconstruction; RCR, rotator cuff repair; OR, odds ratio; SMD, standardized mean difference; LL, lower limit; CI, confidence interval; UL, upper limit; VAS, visual analog scale; ASES, the American Shoulder and Elbow Surgeons score; UCLA, the University of California at Los Angeles score; ROM, range of motion; NA, Not applicable.

## Data Availability

No new data have been created, and all the results obtained are available in this article. Literature searches can be made available on request to the corresponding principal author.

## Supplementary Materials

The following supporting information can be downloaded at: https://www.mdpi.com/article/10.3390/jcm13041052/s1, Figure S1: Sensitivity analysis for ASES score; Figure S2: Forest plot of ASES score before sensitivity analyses; Figure S3: Sensitivity analysis for AHI; Figure S4: Forest plot of AHI score before sensitivity analyses; Table S1: Summary of subgroup analyses according to SCR techniques.
