# Supplementary material for: Outcomes of Superior Capsular Reconstruction Using the Long Head of the Biceps Tendon in Large to Massive Rotator Cuff Tears: A Meta-Analysis and Systematic Review
Source: J Clin Med. 2024 Feb 12;13(4):1052. doi: 10.3390/jcm13041052 (PMC10889218; doi:10.3390/jcm13041052)
Supplement: Supplementary file 1 [file jcm-13-01052-s001.zip › jcm-2833060-supplementary.pdf]

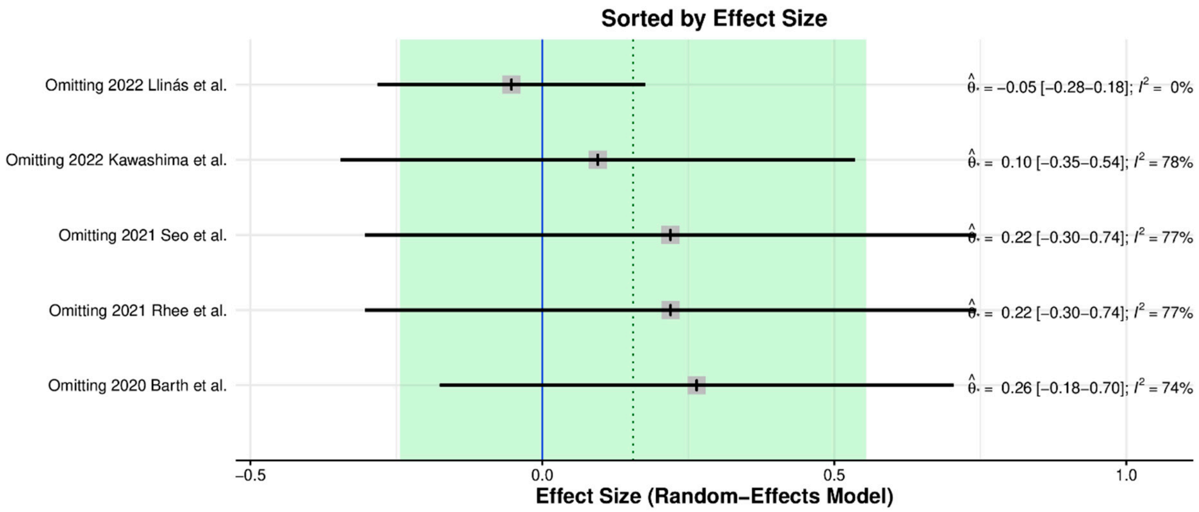

Supplementary Figure S1. Sensitivity analysis for ASES score

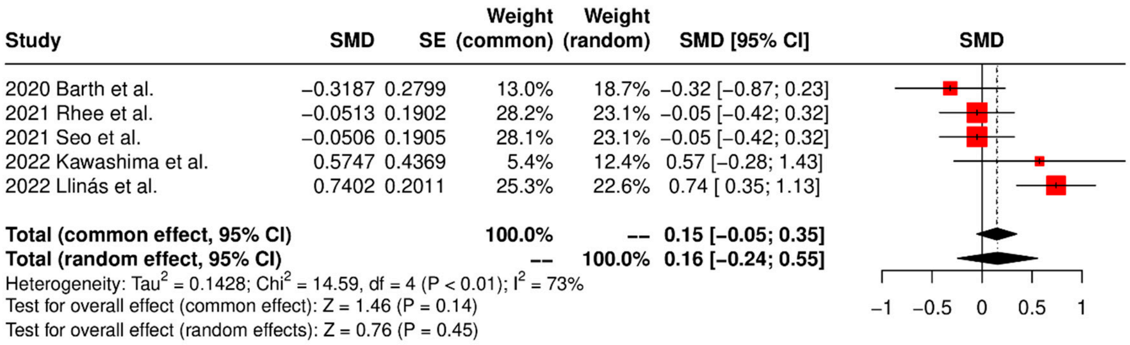

Supplementary Figure S2. Forest plot of ASES score before sensitivity analyses

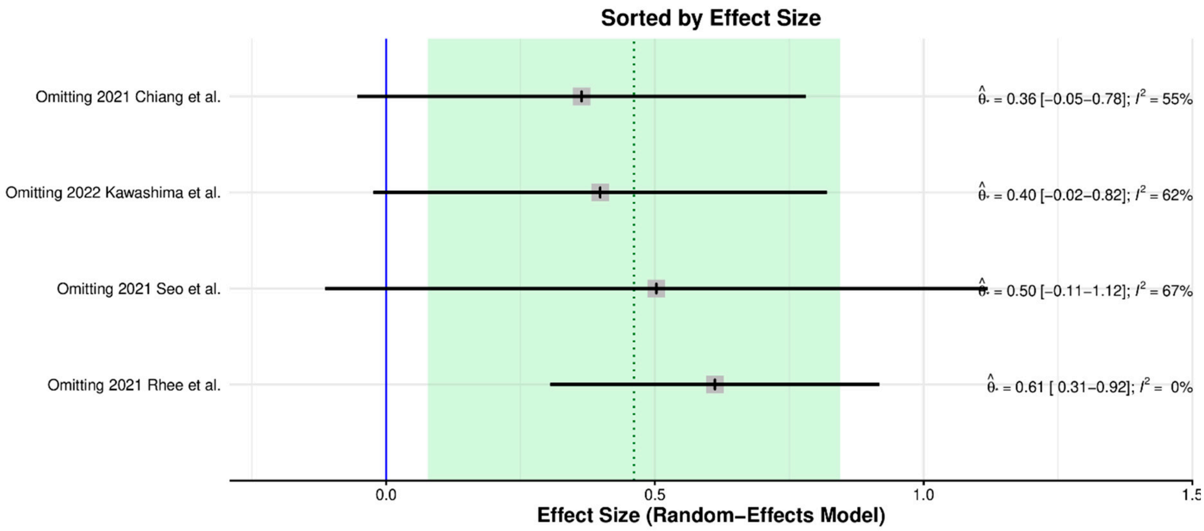

Supplementary Figure S3. Sensitivity analysis for AHI

Study	SMD	SE	Weight (common)	Weight (random)	SMD [95% CI]
2021 Chiang et al.	0.8317	0.3311	13.3%	20.3%	0.83 [0.18; 1.48]
2021 Rhee et al.	0.0385	0.1902	40.3%	33.2%	0.04 [−0.33; 0.41]
2021 Seo et al.	0.4890	0.1930	39.1%	32.9%	0.49 [0.11; 0.87]
2022 Kawashima et al.	0.8693	0.4478	7.3%	13.7%	0.87 [−0.01; 1.75]
**Total (common effect, 95% CI)**			**100.0%**	**--**	**0.38 [0.14; 0.62]**
**Total (random effect, 95% CI)**			**--**	**100.0%**	**0.46 [0.08; 0.84]**

Heterogeneity: $Tau^2 = 0.0793$; $Chi^2 = 6.60$, df = 3 (P = 0.09); $I^2 = 55\%$
Test for overall effect (common effect): Z = 3.15 (P < 0.01)
Test for overall effect (random effects): Z = 2.36 (P = 0.02)

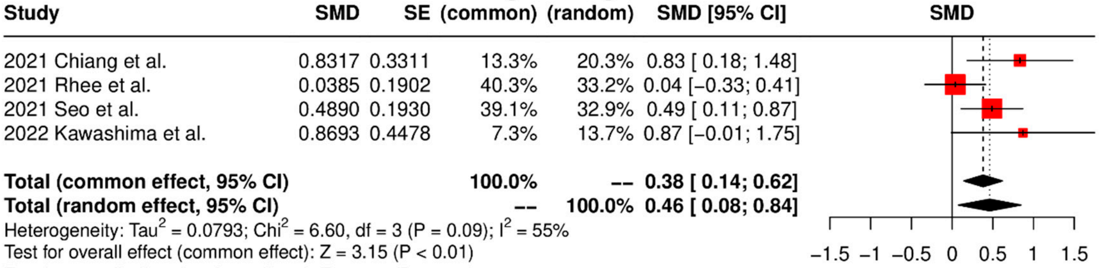

Supplementary Figure S4. Forest plot of AHI score before sensitivity analyses

Supplementary table S1. Summary of subgroup analyses according to SCR techniques

	SCR using LHBT with distal tenotomy					SCR using LHBT without distal tenotomy					Statistical significance
	OR or SMD	LL 95%CI	UL 95%CI	Number of studies	P value	OR or SMD	LL 95%CI	UL 95%CI	Number of studies	*P* value	
Retear rate	0.13	0.05	0.30	3	<0.01	0.28	0.14	0.57	3	<0.01	No difference
ASES score	0.26	-0.39	0.90	2	0.44	0.09	-0.46	0.63	3	0.76	No difference
VAS	-0.31	-0.62	0.01	2	0.06	0.05	-0.21	0.31	2	0.71	No difference
ROM (Forward flexion)	0.22[a]	-0.56	1.00	1	0.58	0.39	-0.04	0.83	3	0.07	No difference
ROM (External rotation)	-0.29	-0.61	0.04	2	0.08	0.03	-0.23	0.29	3	0.82	No difference
Acromiohumeral interval	0.83[a]	-0.01	1.67	1	0.05	0.36	-0.05	0.78	3	0.09	No difference

SCR, superior capsular reconstruction; OR, odds ratio; SMD, standardized mean difference; LL, lower limit; CI, confidence interval; UL, upper limit VAS, visual analog scale; ASES, the American Shoulder and Elbow Surgeons score; ROM, range of motion

[a] Estimates based on one study
